# Role of acetylcholine and polyspecific cation transporters in serotonin-induced bronchoconstriction in the mouse

**DOI:** 10.1186/1465-9921-7-65

**Published:** 2006-04-12

**Authors:** Wolfgang Kummer, Silke Wiegand, Sibel Akinci, Ignatz Wessler, Alfred H Schinkel, Jürgen Wess, Hermann Koepsell, Rainer V Haberberger, Katrin S Lips

**Affiliations:** 1 Institute for Anatomy and Cell Biology, Justus-Liebig-University, 35385 Giessen, Germany; 2 Department of Pathology, University of Mainz, Germany; 3 Division of Experimental Therapy, The Netherlands Cancer Institute, 1066 CX Amsterdam, The Netherlands; 4 Laboratory of Bioorganic Chemistry, National Institute of Diabetes and Digestive and Kidney Diseases, Bethesda, Maryland 20892, USA; 5 Institute for Anatomy and Cell Biology, Julius-Maximilians-University, 97070 Würzburg, Germany; 6 Department of Anatomy and Histology, Flinders University, 50001 Adelaide, Australia

## Abstract

**Background:**

It has been proposed that serotonin (5-HT)-mediated constriction of the murine trachea is largely dependent on acetylcholine (ACh) released from the epithelium. We recently demonstrated that ACh can be released from non-neuronal cells by corticosteroid-sensitive polyspecific organic cation transporters (OCTs), which are also expressed by airway epithelial cells. Hence, the hypothesis emerged that 5-HT evokes bronchoconstriction by inducing release of ACh from epithelial cells via OCTs.

**Methods:**

We tested this hypothesis by analysing bronchoconstriction in precision-cut murine lung slices using OCT and muscarinic ACh receptor knockout mouse strains. Epithelial ACh content was measured by HPLC, and the tissue distribution of OCT isoforms was determined by immunohistochemistry.

**Results:**

Epithelial ACh content was significantly higher in OCT1/2 double-knockout mice (42 ± 10 % of the content of the epithelium-denuded trachea, n = 9) than in wild-type mice (16.8 ± 3.6 %, n = 11). In wild-type mice, 5-HT (1 μM) caused a bronchoconstriction that slightly exceeded that evoked by muscarine (1 μM) in intact bronchi but amounted to only 66% of the response to muscarine after epithelium removal. 5-HT-induced bronchoconstriction was undiminished in M_2_/M_3 _muscarinic ACh receptor double-knockout mice which were entirely unresponsive to muscarine. Corticosterone (1 μM) significantly reduced 5-HT-induced bronchoconstriction in wild-type and OCT1/2 double-knockout mice, but not in OCT3 knockout mice. This effect persisted after removal of the bronchial epithelium. Immunohistochemistry localized OCT3 to the bronchial smooth muscle.

**Conclusion:**

The doubling of airway epithelial ACh content in OCT1/2^-/- ^mice is consistent with the concept that OCT1 and/or 2 mediate ACh release from the respiratory epithelium. This effect, however, does not contribute to 5-HT-induced constriction of murine intrapulmonary bronchi. Instead, this activity involves 1) a non-cholinergic epithelium-dependent component, and 2) direct stimulation of bronchial smooth muscle cells, a response which is partly sensitive to acutely administered corticosterone acting on OCT3. These data provide new insights into the mechanisms involved in 5-HT-induced bronchoconstriction, including novel information about non-genomic, acute effects of corticosteroids on bronchoconstriction.

## Background

Serotonin (5-hydroxytryptamine, 5-HT) causes constriction of murine airways that is sensitive to atropine both in vivo and in vitro [1,2]. This response is markedly reduced after removal of the epithelium in the isolated mouse trachea [3]. Hence, it has been suggested that stimulation of epithelial 5-HT_2A _receptors on mouse tracheal epithelial cells triggers the release of acetylcholine (ACh) from these cells, which then causes airway constriction [3]. In line with this notion, the presence of ACh, its synthesizing enzyme choline acetyltransferase, and of the high-affinity choline transporter, CHT1, that mediates the rate-limiting step of ACh synthesis, has been demonstrated in the airway epithelium of several mammalian species [4-7,3]. It remains unclear, however, by which molecular mechanism ACh is released from airway epithelial cells. In cholinergic neurons, ACh is synthesized in the cytosol by choline acetyltransferase (ChAT), translocated into synaptic vesicles by the vesicular ACh transporter (VAChT) and then released by exocytosis. VAChT expression has been detected in some airway epithelial cells [7,8]. However, since 5-HT-induced constriction of the mouse trachea is insensitive to botulinum toxin A [3], it is unlikely that exocytotic ACh release is involved in this activity. Recently, polyspecific organic cation transporters (OCTs) have emerged as alternative mediators for the release of ACh. All known OCT isoforms (OCT1-3) are expressed by rat and human airway epithelia [8]. OCT inhibitors and pre-treatment with OCT-anti-sense-oligonucleotides diminish ACh release from human placental villi [9]. Recently, we demonstrated that rat and human OCT1 and OCT2 expressed by *Xenopus *oocytes mediate ACh transport, and that this effect could be blocked by corticosteroids [8].

Hence, we speculated that corticosteroid-sensitive OCTs may mediate 5-HT-induced ACh release from airway epithelial cells, thus leading to airway constriction in the mouse. In order to test this hypothesis, 5-HT-induced bronchoconstriction of small intrapulmonary airways and the sensitivity of this response to corticosterone were studied videomorphometrically in precision-cut lung slices (PCLS) [10-12] taken from OCT1-3-deficient mice [13,14]. PCLS offer the advantage to study smallest bronchi whose bronchoconstrictor response can, otherwise, not directly been visualised. The presence of ACh in murine respiratory epithelium was validated by biochemical techniques and ChAT-immunohistochemistry, and we obtained evidence for a significant role of OCT1 and 2 in the release of ACh from airway surface epithelium. The potential involvement of ACh in 5-HT-induced bronchoconstriction was tested by using mice deficient in both M_2 _and M_3 _muscarinic ACh receptors (M2/3R^-/- ^mice). We demonstrated previously that muscarinic agonists are unable to constrict bronchi taken from M2/3R^-/- ^mice [11]. Surprisingly, the data obtained with these mutant strains revealed that ACh is not involved in 5-HT-induced bronchoconstriction. On the other hand, we uncovered a direct involvement of smooth muscular OCT3 in 5-HT-induced bronchoconstriction which proved to be corticosterone-sensitive.

## Methods

### Animals

Lungs were taken from 8–12 wk old male M2/3R^-/- ^mutant mice and M2/3R^+/+ ^wild-type mice of the same genetic background [129/J1 (25 %) × 129SvEv (50 %) × CF1 (25 %)], OCT1/2^-/- ^mice, OCT3^-/- ^mice, and their corresponding wild-type strain (FVB) (all age- and gender-matched). The generation of the mutant mouse strains used in this study has been described previously [11]. M2/3R^-/- ^mice and the corresponding wild-type strain were kept under specified pathogen-free conditions, whereas the remaining animals were kept in a standard animal facility.

### ACh assay

FVB and OCT1/2^-/- ^mice were killed by isoflurane inhalation. Tracheas were carefully cleaned from adhering tissue and fixed in a Petri dish with the luminal surface facing upwards. A cotton-tipped applicator (Q-tip) was gently rubbed along the luminal surface as described earlier [5] and thereafter placed in 1 ml 15% formic acid in acetone (v/v). Epithelium-intact or denuded tracheas were also placed in 1 ml 15% formic acid in acetone (v/v) and minced with scissors. After a 30 min incubation on ice, Q-tips were removed and the extraction medium was centrifuged (2 min; 10 000 rpm), and the supernatant was evaporated to dryness by nitrogen. The dried sample was resuspended in 800 μl of the mobile phase of the HPLC system, and 20 μl were injected.

ACh was measured by cationic exchange HPLC combined with bioreactors and electrochemical detection as described elsewhere [15,4]. The BAS 481 microbore system was used (Bioanalytical Systems Inc., West Lafayette, USA). ACh and choline were separated on an analytical SepStik column (1 × 530 mm; BAS, Axel Semrau, Sprockhövel, Germany) using a mobile phase of 45 mM phosphate buffer and 0.3 mM EDTA (adjusted to pH 8.5). The analytical column was followed by an immobilized enzyme reactor containing acetylcholinesterase to hydrolyze ACh and choline oxidase to produce H_2_O_2 _from the breakdown product choline. H_2_O_2 _flowing across a platinum electrode is oxidized producing a current which is proportional to the amount of ACh in the sample. Twenty μl samples were injected by an automatic injector. The amount of ACh was calculated by comparison with external standard containing 1 pmol/20 μl of both ACh and choline.

### Videomorphometry

PCLS were prepared using a slightly modified version of the protocol described by Martin et al. [10], as reported in full detail earlier [11,12]. Very briefly, mice were killed by cervical dislocation, the pulmonary vasculature was flushed blood-free via the right ventricle, and the airways were filled via the cannulated trachea with low melting point agarose (Sigma, Taufkirchen, Germany). Lungs and heart were dissected in toto, cooled, and PCLS were cut (vibratome VT1000S, Leica, Bensheim, Germany) at a thickness of 200 μm from the left lobe of the lung and incubated in minimal essential medium (MEM; GIBCO, Karlsruhe, Germany) at 37°C for 4–7 h to remove the agarose. Experiments were performed in HEPES-Ringer buffer in a lung slice superfusion chamber (Hugo Sachs Elektronik, March, Germany) mounted on an inverted microscope. Images of bronchi of about 200 μm in diameter were recorded with a CCD camera and analyzed with Optimas 6.5 software (Stemmer Imaging, Puchheim, Germany). Only those bronchi were included in the final data analysis which responded to a test stimulus of 10^-6 ^M muscarine (or, in case of M2/3R^-/- ^mice, 10^-5 ^M U44619, a thromboxane analogue) with a reduction of luminal area of at least 25 %.

Epithelia were removed after preparation of PCLS and wash-out of agarose. PCLS were placed in HEPES-Ringer buffer in a Petri dish on a binocular stage and immobilized with a mesh of nylon strings connected to a platinum ring. Under microscopic control, the lumen of selected bronchi was manually rubbed with a fine steel-needle (0.15 mm diameter; Faber, Berlin, Germany) mounted onto a wooded rod, until the epithelium could be seen floating off. The position of treated bronchi within PCLS was recorded to assure subsequent re-identification. PCLS were returned for 2–8 h into the equilibrium medium in the incubator before the start of the experiments. After completion of the videomorphometric recordings, PCLS were placed on microscopic slides and cover-slipped. The efficiency of epithelium removal was then assessed microscopically. Only those bronchi were included in the analysis in which at least 75 % of the luminal circumference was found to be devoid of epithelial cells. Epithelium denudation of the entire circumference could not be achieved.

Muscarine, atropine, 5-HT, U44619, and corticosterone were purchased from Sigma, Taufkirchen, Germany. Corticosterone was dissolved in ethanol at 10^-2 ^M, and diluted in water to the desired experimental concentration immediately before use.

### Immunofluorescence

*OCTs*. Thoraxes of wild-type FVB mice (n = 5) and OCT1/2^-/- ^mice (n = 3) were dissected, the lungs were filled with Tissue-Tek (Sakura Finetek, Zoeterwoude, Netherlands), and the tissues were shock-frozen in melting isopentane. Cryosections (10 μm) were fixed in acetone for 10 min at -20°C, preincubated for 1 h in phosphate-buffered saline (PBS) containing 50 % horse serum, and then covered for 12–16 h with primary antibodies diluted in PBS. The affinity-purified antibody against OCT1 (dilution 1:20; Alpha Diagnostic, San Antonio, TX, USA) was raised against a 21 amino acid sequence near the C-terminus of rat OCT1, which shares 95 % amino acid identity with mouse OCT1. Two affinity-purified antibodies against OCT2 were used. One was raised against amino acids 533–547 (near the C-terminus) of human OCT2 (dilution 1:100; [8]) that share 82 % amino acid identity with mouse OCT2, and the other one was raised against a 21 amino acid sequence near the C-terminus of rat OCT2 (1:400; Alpha Diagnostic) sharing 76 % amino acid identity with mouse OCT2. The affinity-purified antibody against OCT3 was raised against amino acids 297–313 of human OCT3 (dilution 1: 500; [8]) that share 82 % identity with mouse-OCT3. Since the OCT3 antibody apparently labelled smooth muscle cells, it was also applied in combination with a mouse monoclonal marker antibody for this cell type, i.e. anti-α-smooth muscle actin antibody directly conjugated to fluorescein-isothiocyanate (clone 1A4; Sigma, Taufkirchen, Germany; dilution 1:500) to ascertain muscular localization. After washing in PBS, the sections were incubated for 1 h at room temperature with Cy3-coupled donkey anti-rabbit IgG (1:2000 in PBS diluted; Chemicon, Hofheim, Germany) and cover-slipped with carbonate-buffered glycerol (pH 8.6). The sections were evaluated by epifluorescence microscopy (BX60, Olympus, Hamburg, Germany) or with a confocal laser scanning microscope (TCS SP2; Leica, Mannheim, Germany).

We have recently demonstrated the specificity of the primary antibodies in OCT1-3 overexpressing cell lines [8]. On the present material, it was further validated by (a) omission of the primary antibody, (b) preabsorption with the corresponding antigen (40 μg/ml) for 1 h at room temperature prior to use in immunofluorescence, and (c) evaluation of immunofluorescence in OCT-deficient mice.

*ChAT*. Lungs from 4 FVB mice were prepared as described above. Cryosections (10 μm) were dipped in phosphate-buffered 15 % picric acid/2 % paraformaldehyde, preincubated for 1 h in PBS containing 0.5 % Tween 20 (Sigma) and 0.1 % bovine serum albumin (Sigma), and covered overnight with a rabbit antiserum (dilution 1:8000) raised against a synthetic peptide corresponding to amino acids 282–295 of the predicted rat ChAT protein [16]. This antiserum specifically recognizes the "common type" of ChAT [16]. After PBS washes, the sections were incubated for 1 h at room temperature with Cy3-coupled donkey anti-rabbit IgG (1:1000; Chemicon), postfixed for 10 min in 4 % buffered paraformaldehyde, washed, and cover-slipped with carbonate-buffered glycerol (pH 8.6). Micropgraphs were taken by confocal laser scanning microscopy.

Control sections were incubated with antiserum that had been preincubated with its corresponding peptide (20 μg/ml) for 1 h at room temperature prior to use in immunofluorescence.

### Statistical analysis

Data are presented as mean ± standard error of the mean. Non-matched groups were compared by Mann-Whitney U-test. In case of more than two groups, analysis was done first by global Kruskal-Wallis rank sum test, and if significant (p < 0.05) differences were observed, comparison between two groups was made by Mann-Whitney U-test. Throughout, differences were considered as statistically significant when p < 0.05.

## Results

### ACh in murine trachea and respiratory epithelium

We used an HPLC procedure to determine ACh levels separately in epithelium and underlying tissues in wild-type (FVB strain) and OCT1/2^-/- ^mice. Using wet weight of the sample as reference, ACh content of the epithelium-denuded trachea was not significantly different in these strains (FVB: 17.34 ± 4.07 pmol/mg; n = 11; OCT1/2^-/-^: 15.90. ± 4.0 pmol/mg, n = 9). The relative proportion of epithelial ACh, however, was significantly (p < 0.01) higher in OCT1/2^-/- ^mice (42 ± 10 % of that in the denuded specimens) than in corresponding wild-type (FVB) mice (16.8 ± 3.6 %). In a few additional samples, tracheal specimens with intact epithelium were analysed, yielding 36.5 ± 4.4 pmol/mg in FVB mice (n = 4) and 28.5 ± 3.50 pmol/mg in OCT1/2^-/- ^mice (n = 3).

**Figure 1:**
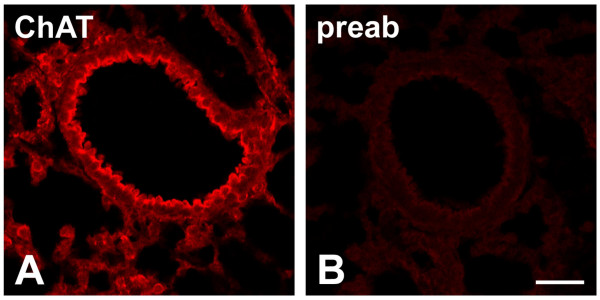
Immunohistochemical localization of ChAT in murine peripheral bronchi. Respiratory epithelial cells are strongly ChAT-immunoreactive in wild-type FVB mice (A). The specificity of this labelling is indicated by its absence after preabsorption of the antiserum with its corresponding antigenic peptide (B). *Bar *represents 50 μm.

Bronchi of about 200 μm in diameter were too small to dissect the respiratory epithelium for biochemical ACh analysis. The ACh synthesizing enzyme, ChAT, was demonstrated in epithelial cells of these bronchi by immunohistochemistry (Fig. 1).

### Role of the epithelium and of ACh in 5-HT-induced bronchoconstriction

**Figure 2:**
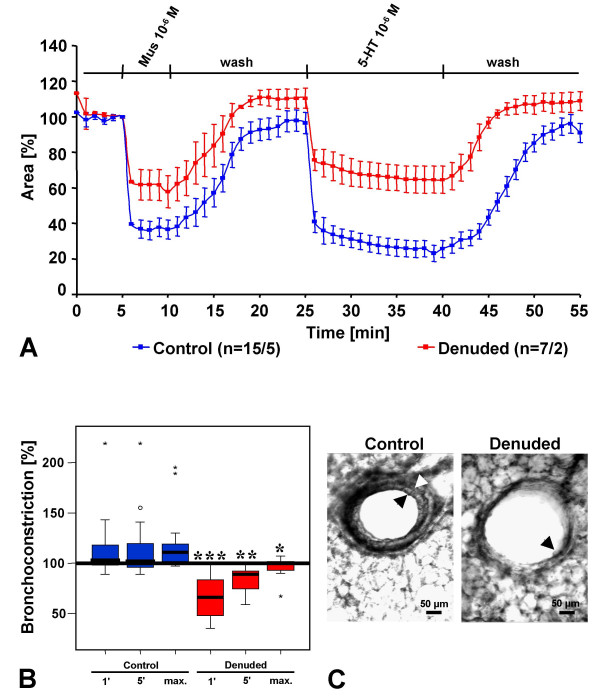
Effect of epithelium removal on constriction of peripheral bronchi in PCLS of M2/3R^+/+ ^mice. (A) Reduction of luminal area of intact (control, *blue*) and epithelium-denuded (denuded, *red*) peripheral bronchi in response to muscarine (Mus, 10^-6 ^M) and 5-HT (10^-6 ^M). The numbers in parentheses refer to the numbers of bronchi/number of lungs from which they were taken. Panel (B) illustrates the magnitude of the response to 5-HT (10^-6 ^M) compared to that to muscarine (10^-6 ^M) which was set as 100 %. Control bronchi react slightly stronger to 5-HT than to muscarine, whereas the 5-HT response is significantly smaller that the muscarine response after epithelium removal, particularly at 1 min (1') after agonist application. The box plots shows percentiles 0, 25, 50 (median), 75, and 100; individual data points beyond 3× S.D. are indicated by * or °. ***p < 0.001, **p < 0.01, *p < 0.05 (comparison of corresponding time points by Mann-Whitney U-test). (C) Microscopic appearance of control and epithelium-denuded bronchi. In the left panel, *arrowheads *indicate thickness of the epithelial layer in a control bronchus. In the right panel, the *arrowhead *points to a small remnant of epithelium after mechanical denudation of the epithelium.

Small intrapulmonary bronchi from M2/3R^+/+ ^wild-type mice strongly constricted in response to both muscarine (10^-6 ^M) and to 5-HT (10^-6 ^M; Fig. 2). The magnitude of the 5-HT-induced bronchoconstriction even surpassed that evoked by muscarine (Fig. 2). Mechanical (partial) removal of the epithelium diminished the constriction to muscarine (Fig. 2), consistent with the results of a previous study involving the chemical (Triton X-100) ablation of the murine tracheal epithelium [3]. Removal of the airway epithelium also led to a significant reduction in the 5-HT-induced bronchoconstriction response (Fig. 2). However, removal of the epithelium had a more pronounced effect on 5-HT- than on muscarine-induced bronchoconstriction. Thus, in contrast to intact bronchi, the magnitude of the 5-HT response was smaller than that evoked by muscarine after epithelium removal.

**Figure 3:**
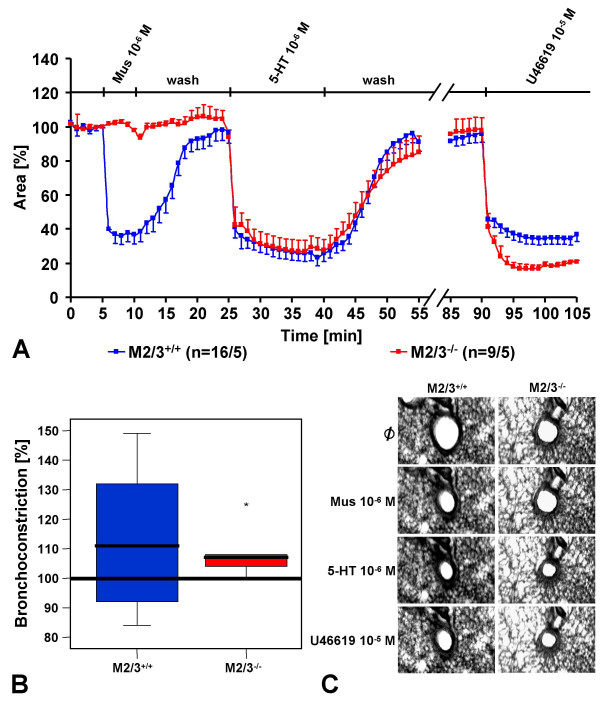
Changes in luminal area of peripheral bronchi in response to muscarine (Mus, 10^-6 ^M), 5-HT (10^-6 ^M), and U44619 (10^-5 ^M) in wild-type (M2/3R^+/+^) and M2/3R^-/- ^mice. (A) 5-HT induces similar responses in both strains. The numbers in parentheses refer to the numbers of bronchi/number of lungs from which they were taken. Panel (B) expresses the 5-HT-induced constriction in percent of that evoked by U44619 in the first min after agonist application. The box plots shows percentiles 0, 25, 50 (median), 75, and 100; * indicates an individual data point beyond 3× S.D. (C) Original images of a peripheral bronchus of a wild-type and an M2/3R^-/- ^double-knockout animal before and after agonist application. As depicted in (A), there is no constriction in response to muscarine in M2/3R^-/- ^mice. On the other hand, both strains show identical responses to 5-HT and U44619.

Bronchi from M2/3R^-/- ^mice were entirely unresponsive to muscarine (10^-6 ^M; Fig. 3), as reported earlier [11]. In striking contrast, 5-HT (10^-6 ^M) induced indistinguishable bronchoconstrictor responses in M2/3R^-/- ^mutant and M2/3R^+/+ ^wild-type mice, both in absolute values and expressed as percent response evoked by the thromboxane analogue, U46610 (10^-5 ^M) (Fig. 3).

**Figure 4:**
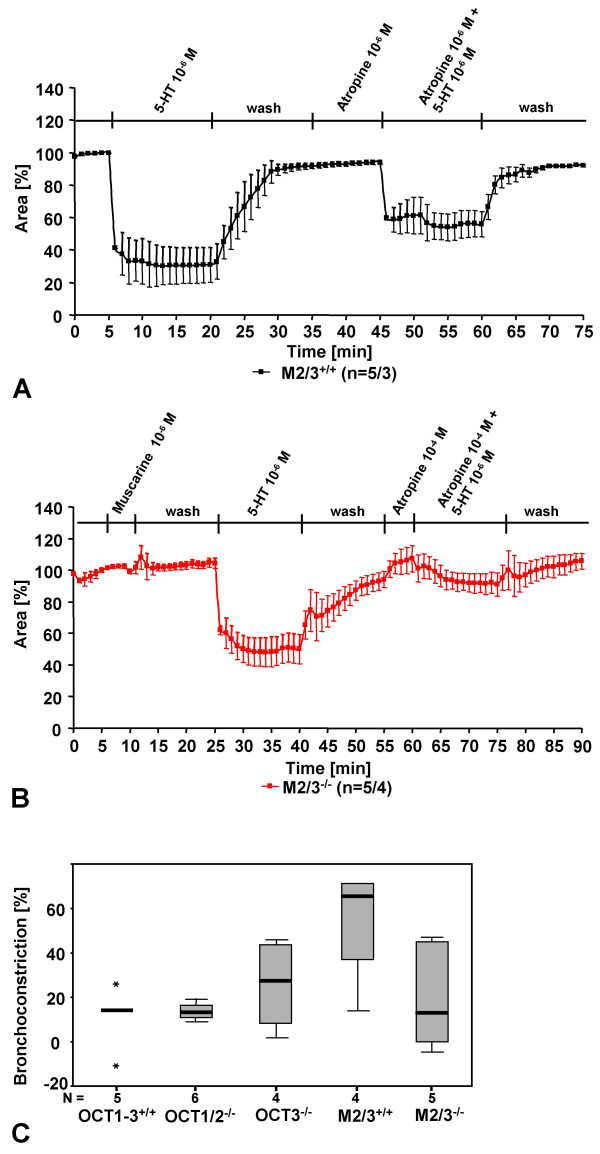
Effect of atropine on 5-HT-induced bronchoconstriction (reduction of bronchial luminal area) in PCLS. Atropine blocks 5-HT-induced constriction partially at 10^-6 ^M (A), and nearly completely at 10^-4 ^M, even in absence of both M_2 _and M_3 _muscarinic receptors (B). The numbers in parentheses refer to the numbers of bronchi/number of lungs from which they were taken. (C) Persisting bronchoconstriction in response to 5-HT (10^-6 ^M) in the presence of 10^-4 ^M atropine in different wild-type and knockout strains. The initial 5-HT-induced bronchoconstriction was set as 100 %.

In preparations from M2/3R^+/+ ^wild-type mice, atropine (10^-6 ^M) partially inhibited 5-HT-induced constriction (Fig. 4A). The same concentration of atropine fully blocked muscarine-induced bronchoconstriction (data not shown, see our previous report [11]). At a higher concentration (10^-4 ^M), however, atropine reduced 5-HT-induced bronchoconstriction by approximately 80 % in all strains tested, including M2/3R^-/-^, OCT1/2^-/-^, OCT3^-/-^, and corresponding wild-type mice (Fig. 4B, C).

### Distribution of OCTs in murine bronchi

**Figure 5:**
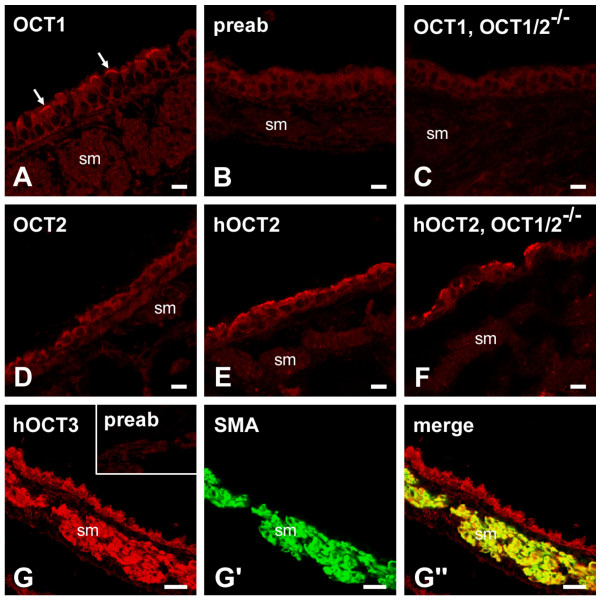
Immunohistochemical localization of OCTs in murine bronchi. OCT1-immunolabelling is localized to the apical membrane of ciliated epithelial cells in wild-type FVB mice (*arrows *in A). The specificity of this labelling is indicated by its absence after preabsorption of the antiserum with its corresponding antigenic peptide (B) and the lack of labelling in OCT1/2^-/- ^mice (C). Neither of the two OCT2-antibodies used in this study showed specific labelling of mouse bronchi (D, E). The spotty labelling of epithelial cells observed with the OCT2-antibody raised against the human sequence (E) was also observed in OCT1/2^-/- ^mice (F), indicating that this signal is non-specific. Specific OCT3-immunolabelling, documented by its absence in the preabsorption control (inset in G), is observed primarily on the bronchial smooth muscle (sm) and, less intensely, on epithelial cells (G). OCT3-localization in smooth muscle cells is confirmed by double-labelling immunofluorescence with OCT3-antibody and a monoclonal antibody against α-smooth muscle actin (SMA) (G') yielding the yellow signal in the merged image (G'). *Bar *represents 10 μm in A-F and 20 μm in G-G".

Immunohistochemistry revealed OCT1-immunoreactivity in the apical membrane of ciliated cells (Fig. 5A). This labelling was OCT1-specific since it was absent when the antiserum was preabsorbed with the corresponding antigenic peptide and when tissue from OCT1/2^-/- ^mice was used for immunohistochemistry (Fig. 5B, C). No specific OCT2-immunolabelling was observed in the bronchial wall (Fig. 5D–F). Specific OCT3-immunoreactivity was most intense in the bronchial smooth muscle and weaker on epithelial cells (Fig. 5G–G").

### Role of OCTs in 5-HT-induced bronchoconstriction

**Figure 6:**
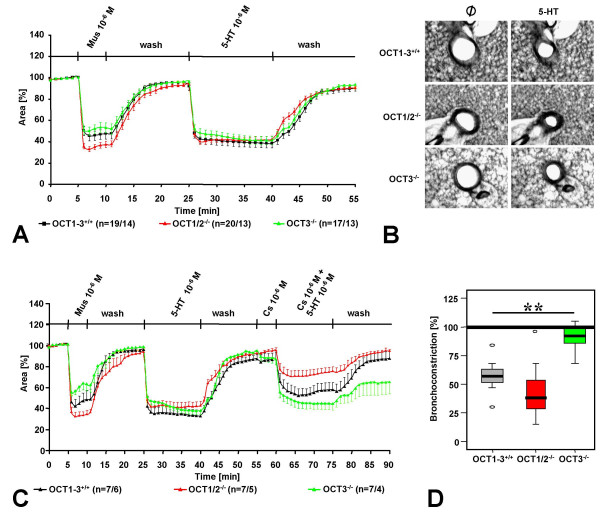
5-HT-induced reduction of bronchial luminal area (bronchoconstriction) in OCT-deficient mice and sensitivity of this response to corticosterone. (A, B) Wild-type FVB mice (OCT1-3^+/+^), OCT1/2^-/- ^mice and OCT3^-/- ^mice exhibit no differences in their response to 5-HT (10^-6 ^M). The numbers in parentheses refer to the numbers of bronchi/number of lungs from which they were taken. (C, D) In wild-type and OCT1/2^-/- ^mice, but not in OCT3^-/- ^mice, the bronchoconstriction in response to 5-HT is significantly reduced by corticosterone (Cs, 10^-6 ^M). Panel (D) depicts the bronchoconstrictor response to 5-HT (10^-6 ^M, 1 min after administration) in the presence of corticosterone (10^-6 ^M), as compared to the response to 5-HT alone (set as 100%). **p < 0.01, Mann-Whitney U-test.

Small intrapulmonary bronchi from OCT1/2^-/-^, OCT3^-/-^, and OCT1-3^+/+ ^wild-type mice reacted with a strong constriction to muscarine (10^-6 ^M) and to 5-HT (10^-6 ^M) (Fig. 6A, B). The absence of OCT1/2 or OCT3 had no significant effect on the 5-HT bronchoconstrictor response. Corticosterone (10^-6 ^M) significantly reduced the 5-HT-induced bronchoconstriction both in wild-type and in OCT1/2^-/- ^mice but was ineffective in OCT3^-/- ^mice (Fig. 6C, D). The effect of epithelium removal on the inhibitory action of corticosterone on 5-HT-induced bronchoconstriction was investigated in M2/3R^+/+ ^wild-type mice. In intact bronchi from this strain, 86 ± 5 % (mean ± S.E.M.; 7 PCLS from 7 lungs) of the 5-HT-induced contraction remained in the presence of corticosterone, so that the corticosterone effect was not as marked as in OCT1-3^+/+ ^wild-type (FVB) mice. This small, but significant reduction of 5-HT-induced contraction by corticosterone in M2/3R^+/+ ^wild-type mice was still present after epithelium removal (remaining contraction: 72 ± 5 %; mean ± S.E.M.; 7 PCLS from 7 lungs).

## Discussion

The present data clearly demonstrate an epithelium-dependent component of 5-HT-induced bronchoconstriction in the mouse, consistent with the results of a previous study on the mouse trachea [3]. It has been suggested that this activity is dependent on the release of ACh from airway epithelial cells [3]. In the *Xenopus *oocyte expression system, both OCT1 and 2, but not OCT3, proved to be able to translocate ACh across the plasma membrane [8]. In the present study, we found that the airway epithelial ACh content was twice as high in OCT1/2^-/- ^than in wild-type mice. This observation supports the concept that OCT1/2 may also play a role in the release of ACh from airway epithelia. However, to our surprise, the magnitude of 5-HT-induced bronchoconstrictor responses was unchanged in PCLS preparations from OCT1/2^-/- ^mice, indicating that 5-HT-induced bronchoconstriction does not require the presence of OCT1 and 2. Moreover, videomorphometric studies showed that PCLS from M2/3R^-/- ^mice remained fully responsive to 5-HT. In contrast, PCLS from M2/3R^-/- ^mice do no longer show a bronchoconstrictor response following cholinergic stimulation, as shown in this and in an earlier study [11]. These data clearly indicate that the release of epithelial ACh is not involved in the 5-HT-induced bronchoconstrictor response, but that another epithelium-derived constrictory factor contributes to this activity.

In previous studies, ACh emerged as a candidate for mediating 5-HT-induced airway constriction in the mouse because this effect could be inhibited by atropine [1-3]. In the present study, we found a large reduction of 5-HT-induced bronchoconstriction only after application of an unusually high concentration of atropine (10^-4 ^M). On the other hand, a much smaller concentration of atropine (10^-6 ^M) was sufficient to fully block muscarine-induced bronchoconstriction. Interestingly, Eum et al. [2] also did not observe a significant inhibition of 5-HT-induced contraction of the isolated mouse trachea at 10^-6 ^M atropine. The inhibition of 5-HT-induced bronchoconstriction by 10^-4 ^M atropine persisted in M2/3R^-/- ^mice, clearly indicating that this high concentration of atropine inhibits airway smooth muscle contractility via non-specific effects that are not due to muscarinic receptor blockade. Indeed, atropine has been described as a competitive antagonist at the 5-HT_3_-receptor [17]. Taken together, the present data demonstrate that 5-HT releases an epithelium-derived bronchoconstrictory factor that is OCT-independent and different from ACh.

We made the striking observation that corticosterone exerted an acute inhibitory effect on 5-HT-induced bronchoconstriction. This acute effect of corticosterone was mediated by OCT3, as demonstrated by its absence in OCT3^-/- ^mice. This finding is of potential clinical relevance since rapid therapeutical effects of a bolus of inhaled glucocorticoids have been reported in asthmatic patients where they reverse airway subsensitivity to β2-agonists [18,19]. In our model, the inhibitory action of corticosterone on 5-HT-induced bronchoconstriction is epithelium-independent since it persisted after epithelium removal. In line with this observation, immunohistochemistry demonstrated that OCT3 is located directly on bronchial smooth muscle cells. In principle, all OCT isoforms tested so far are sensitive to corticosteroids that are not substrates for transport by themselves but inhibit transport of other substances [20]. OCT3, which we identified as being responsible for the acute inhibitory effect of corticosterone on 5-HT-induced bronchoconstriction, has the highest affinity for corticosteroids [20]. It also clears monoamines, including catecholamines and 5-HT, from the extracellular space [21], and hence its blockade is expected to increase the extracellular concentrations of these agents. Indeed, acute human bronchial vasoconstriction elicited by corticosteroids has been explained by inhibition of OCT3 with subsequent rise of extracellular noradrenaline and prolonged activation of α1-adrenoreceptors [22]. However, a separate, specific serotonin transporter (SERT) is highly expressed in the lung [23,24]. As a result, deficiency or blockade of OCT3 may have little impact on 5-HT turnover. In agreement with this notion, the magnitude of the bronchoconstrictor response to 5-HT remained unchanged in bronchi from OCT3^-/- ^mice and the 5-HT response was reduced rather than augmented by corticosterone. It is therefore unlikely that the observed OCT3-mediated inhibition of 5-HT-induced bronchoconstriction by acutely administered corticosterone involves direct interference with 5-HT transport. In view of the electrogenic properties of OCTs [20], the acute inhibitory effect of corticosterone on 5-HT-induced bronchoconstriction might be caused by modulation of membrane potential, but the underlying signal transduction cascade still awaits to be clarified.

## Conclusion

5-HT-induced constriction of murine intrapulmonary bronchi involves two independent pathways. One pathway is dependent on the release of an epithelium-derived constrictory factor that is different from ACh. The second pathway involves the direct stimulation of bronchial smooth muscle cells. This latter pathway is partly sensitive to acutely administered corticosterone acting on OCT3. These data provide new insights into the mechanisms involved in 5-HT-induced bronchoconstriction, including novel information about non-genomic, acute pulmonary effects of corticosteroids.

## Competing interests

The author(s) declare that they have no competing interests.

## Authors' contributions

WK carried out the epithelium removal, evaluated immunohistochemistry, participated in the design of the study and drafted the manuscript. SW carried out epithelium removal, videomorphometric and statistical analyses. SA carried out videomorphometric and statistical analyses. IW performed the ACh assay and revised the manuscript critically for important intellectual content. AHS provided OCT-deficient mice and revised the manuscript critically for important intellectual content. JW provided M2R/M3R-deficient mice and revised the manuscript critically for important intellectual content. HK provided antibodies, added to the design of the study and revised the manuscript critically for important intellectual content. RVH coordinated the videomorphometric setup and breeding of genetically deficient mice strains, and revised the manuscript critically for important intellectual content. KSL performed and evaluated immunohistochemistry, and participated in the design of the study and drafting of the manuscript. The data presented in this manuscript are part of the doctoral thesis of SA.
